# Rapid Lipid Content Screening in *Neochloris oleoabundans* Utilizing Carbon-Based Dielectrophoresis

**DOI:** 10.3390/mi12091023

**Published:** 2021-08-27

**Authors:** Cynthia M. Galicia-Medina, Matías Vázquez-Piñón, Gibran S. Alemán-Nava, Roberto C. Gallo-Villanueva, Sergio O. Martínez-Chapa, Marc J. Madou, Sergio Camacho-León, Jonathan S. García-Pérez, Diego A. Esquivel-Hernández, Roberto Parra-Saldívar, Víctor H. Pérez-González

**Affiliations:** 1 Tecnologico de Monterrey, Escuela de Ingeniería y Ciencias, Ave. Eugenio Garza Sada 2501, Monterrey 64849, Mexico; cynthia.galicia.medina@gmail.com (C.M.G.-M.); matias.vazquez@tec.mx (M.V.-P.); gibran.aleman@gmail.com (G.S.A.-N.); rgallo@tec.mx (R.C.G.-V.); smart@tec.mx (S.O.M.-C.); sergio.camacho@tec.mx (S.C.-L.); garcia.saul@live.com (J.S.G.-P.); 2 Department of Mechanical and Aerospace Engineering, University of California, 4200 Engineering Gateway, Irvine, CA 92697, USA; mmadou@uci.edu; 3 Departamento de Biología Celular, Facultad de Ciencias, Universidad Nacional Autónoma de México, Ciudad de México 04510, Mexico; diegoibt27@gmail.com

**Keywords:** dielectrophoresis, microalgae, biofuel, microfluidics, carbon-microelectromechanical systems

## Abstract

In this study, we carried out a heterogeneous cytoplasmic lipid content screening of Neochloris oleoabundans microalgae by dielectrophoresis (DEP), using castellated glassy carbon microelectrodes in a PDMS microchannel. For this purpose, microalgae were cultured in nitrogen-replete (N+) and nitrogen-deplete (N−) suspensions to promote low and high cytoplasmic lipid production in cells, respectively. Experiments were carried out over a wide frequency window (100 kHz–30 MHz) at a fixed amplitude of 7 V_PP_. The results showed a statistically significant difference between the dielectrophoretic behavior of N+ and N− cells at low frequencies (100–800 kHz), whereas a weak response was observed for mid- and high frequencies (1–30 MHz). Additionally, a finite element analysis using a 3D model was conducted to determine the dielectrophoretic trapping zones across the electrode gaps. These results suggest that low-cost glassy carbon is a reliable material for microalgae classification—between low and high cytoplasmic lipid content—through DEP, providing a fast and straightforward mechanism.

## 1. Introduction

Microalgae have several advantages over terrestrial crops; therefore, they have drawn the attention of the scientific community. Microalgae can grow in non-arable lands using wastewaters [1] and feature a higher CO_2_ fixation rate than terrestrial crops [2]. Moreover, microalgae produce many high-value compounds (e.g., omega-3 and omega-6 fatty acids) and have been proven to be excellent biomass sources for biofuel production [3,4]. However, the high harvesting cost is the main limitation for microalgae mass production, with it representing 20–30% of the total production cost [5].

One way to reduce microalgae harvesting costs is to determine their optimal harvesting time through lipid content monitoring—which also allows for the identification of high-producing cell strains [6]. Several approaches have been developed to determine cell lipid content, including solvent extraction [7], gravimetric determination [8], gas chromatography [9], and high-performance liquid chromatography [10]. Unfortunately, these techniques are expensive and time-consuming, requiring numerous pre-analysis steps and large biomass volumes [11,12,13]. Therefore, alternative efficient cell lipid content monitoring techniques are constantly sought after.

Dielectrophoresis (DEP) is an electrokinetic phenomenon used to manipulate (e.g., filter, separate, concentrate) polarizable particles suspended in a fluid when subjected to a spatially non-uniform electric field [14]. The DEP response of a particle depends on its shape and size, its dielectric properties (i.e., permittivity and electrical conductivity) and those of the suspending medium, and the frequency and spatial non-uniformity of the applied electric field. Because the dielectric properties of microalgae rely on their cytoplasmic lipid content, their DEP response to frequencies larger than 20 MHz was recently studied [15]. Additionally, viable and non-viable cells were separated [16], the effect of different suspending medium conductivities was analyzed [17], and cells were manipulated under continuous medium flow [18].

Two main types of DEP-driven microfluidic devices exist: insulator-based DEP (iDEP) and electrode-based DEP (eDEP). Focusing on monitoring cell lipid content, most iDEP devices are restricted to working under direct current (DC) voltages (i.e., neglecting the influence of cell permittivity in the DEP response) and all require stimulations above 100 V [19]. In eDEP, voltage requirements are lower and alternating current (AC) stimulations can be used. Nonetheless, the fabrication of metallic [20] and electroconductive polymeric [21] electrodes is expensive and cumbersome. Moreover, metal electrodes require a metallic adhesion layer that forms a galvanic couple when in contact with an electrolyte, leading to electrode corrosion and sample contamination [22]. The fabrication of glassy carbon (GC) electrodes is more straightforward [23] and the resulting material has an adequate electrical conductivity and a wider electrochemical stability window than most metals—decreasing the likelihood of electrolysis and sample contamination [24].

GC is produced by the thermochemical decomposition of a photoresist deposited on a substrate. The resulting material has proven to be versatile for different microscale functionalities, and has been extensively characterized [22,25,26] and successfully utilized as an electrode material for biosensing [27,28], energy storage [29,30], and electrokinetic [31,32,33] applications. Moreover, several post-processing techniques have been developed to improve the functionality, performance, and reproducibility of GC as an electrode material [34,35,36].

In this work, the DEP behavior of microalgae with high and low lipid contents (HLC and LLC, respectively) was studied using a GC interdigitated electrode array (IDEA): Carbon-DEP. For this process, two microalgae samples were grown under different N_2_ conditions. The variation in their lipid content was assessed to assure a statistically significant difference among them. Then, a finite element analysis (FEA) was conducted to determine the most favorable IDEA geometry, based on the intensity of the non-uniform electric field across the microchannel. The resulting design was fabricated, and experiments were carried out on a wide frequency spectrum (100 kHz–30 MHz). The results were contrasted among cell types (LLC vs. HLC) under the same stimulation conditions.

## 2. Theoretical Background

When a polarizable particle suspended in a fluid is subjected to an electric field, an effective dipole moment is induced in it. If the electric field is spatially non-uniform, a dielectrophoretic force ($F_{DEP}$), whose magnitude and direction depend on the polarizability of the particle relative to that of the suspending medium, will act on the particle. In positive DEP (pDEP), the particle is more polarizable than the medium and it moves towards regions of high field intensity (i.e., in the direction of $\nabla \left(E\cdot E\right)$). In negative DEP (nDEP), the particle is less polarizable than the medium and it moves away from regions of high field intensity (i.e., in the opposite direction of $\nabla \left(E\cdot E\right)$) [37]. The DEP force acting on a spherical polarizable particle is defined as:

(1) FDEP=πa3εmε0RefCM∇E⋅E

where, a is the particle radius, $\varepsilon_{m}$ is the relative permittivity of the suspending medium, $\varepsilon_{0}$ is the dielectric constant in vacuum, and $f_{CM}$ is the frequency-dependent Clausius–Mossotti factor, which is expressed in terms of complex permittivities:

(2) $$f_{CM}=\frac{\varepsilon_{p}^{*}-\varepsilon_{m}^{*}}{\varepsilon_{p}^{*}+2\varepsilon_{m}^{*}}$$

with $\varepsilon_{p}^{*}$ and $\varepsilon_{m}^{*}$ representing the complex permittivities of the particle (*p*) and the medium (*m*). Complex permittivities are given by:

(3) $$\varepsilon_{i}^{*}=\varepsilon_{0}\varepsilon_{i}-j\frac{\sigma_{i}}{\omega }$$

where i=p,m, $\varepsilon_{i}$ is the relative permittivity and $\sigma_{i}$ is the conductivity; ω=2πf is the angular frequency of the input voltage, and $j=\sqrt{-1}$ is the imaginary unit [38].

## 3. Materials and Methods

### 3.1. Device Design, Fabrication, and Characterization

Equation (1) was solved for $\nabla \left(E\cdot E\right)$ to determine the minimum magnitude required to induce a significant DEP force on cells with spherical geometry and 4 µm of diameter (input data obtained from [39]). We designed a rectangular castellated IDE array to generate $\nabla \left(E\cdot E\right)$ with a higher magnitude than the calculated minimum and low-amplitude voltages [40]. According to models built in COMSOL Multiphysics (COMSOL Inc., Burlington, MA, USA), an IDEA formed by two combs—each with 38 20-μm-wide fingers and featuring 15 μm gaps between opposite electrodes—provides an excellent pDEP cell-trapping performance (see Section 4.2 for details).

**Figure 1:**
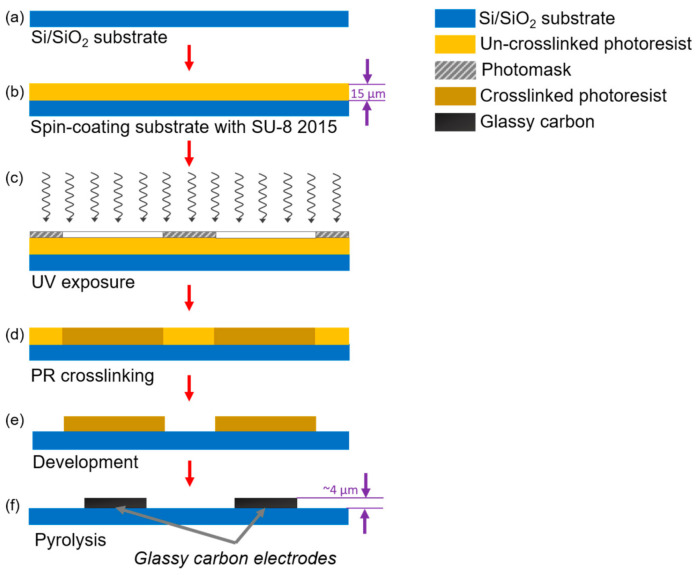
Standard photolithography process followed to fabricate castellated interdigitated electrode array. Here, (**a**) a silicon wafer is used as a substrate for the structures. (**b**) A spin coating process is carried out to deposit a 15 μm layer of PR. Then, (**c**) the electrodes’ geometry is patterned through a photomask using UV light. (**d**) A hot bake is used to crosslink unexposed PR. (**e**) The crosslinked material is then developed using a PR developer solution; finally, (**f**) the crosslinked PR is pyrolyzed at 900 °C to release non-carbon elements from the structure and obtain the glassy carbon electrodes.

A standard photolithography process was used to fabricate the IDEA, as detailed elsewhere [41]. In brief, a 4-inch silicon wafer with a 500-nm-thick SiO_2_ layer (Noel Technologies, Campbell, CA, USA) was spin-coated with SU-8 2015 photoresist (PR) (MicroChem Corp, Westborough, MA, USA) to a thickness of 15 μm (Figure 1a,b). After soft baking at 95 °C for 3 min to clear the PR from solvents, the electrode geometries were patterned by UV exposure at 140 mJ/cm^2^ through a thin-film photomask and baked again for 3 min to complete crosslinking (Figure 1c,d). Finally, the crosslinked structures were developed for 3 min using SU-8 developer (MicroChem, Newton, MA, USA) (Figure 1e). To produce the GC material from the patterned PR, the structures were carbonized in a tube furnace (RD-M, R.D. Webb, Natick, MA, USA) under an N_2_ flow of 2000 sccm at 900 °C for 1 h (Figure 1f). The temperature profile employed in this work was set as previously reported for a standard Carbon-MEMS process [41].

For experimental testing, a poly(dimethylsiloxane) (PDMS) rectangular microchannel (Sylgard 184; Dow Corning, MI, USA) was fabricated by soft-lithography using a 10:1 base-to-curing agent ratio, as recommended by the manufacturer. The channel dimensions are 75 μm-height, 1 mm-width, and 1.5 cm-length. The PDMS slab was bonded to the SiO_2_/GC device by oxygen plasma treatment (PDC-001, Ithaca, NY, USA). Copper wires were soldered to the carbon electrodes and connected to a waveform generator (DS345, Stanford Research Systems, Sunnyvale, CA, USA) to provide the stimulation signal.

**Figure 2:**
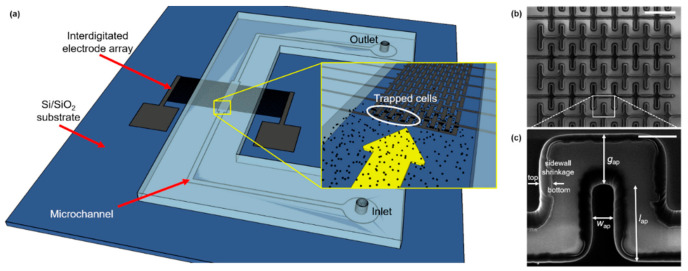
(**a**) Schematic diagram of the Carbon-DEP microfluidic device. The microfluidic channel is connected to inlet and outlet reservoirs, and the Carbon IDEA is powered by an external waveform generator (not shown) across pads. Inset shows a close view of the IDEA with microalgae cells flowing through the microchannel. (**b**) SEM image of the electrode array (scale bar: 100 μm), and (**c**) close view of the pDEP trapping region (scale bar: 20 μm). Effective dimensions after pyrolysis (ap) shown in (**b**): gap between opposite electrodes, *g*_ap_ = 25.45 µm; width, *w*_ap_ = 9.91 µm; length, *l*_ap_ = 38.15 µm. Sidewall shrinkage can be noticed along the electrode height from the original SU-8 boundary (bottom) to the final GC edge (top).

As shown in Figure 2b,c, the castellated IDEA shrunk considerably in volume during pyrolysis. This effect is observed due to the non-carbon elements comprising the photoresist material being released to the ambient environment due to the high temperature and absence of oxygen in the tube furnace. The remaining structure is composed of only the carbon backbone of photoresist molecules; thus, a conformal shrinking is observed. For the Carbon-MEMS process used in this work, a volumetric shrinkage between 40% and 90% was expected, which depends on the aspect ratio of the polymeric structures [42,43]. In this specific case, the volumetric shrinkage led to a decrease in the electrodes’ width of ~50%, causing, in turn, an increase in the gap between opposite IDEAs of ~10 µm. Sidewall shrinkage can be clearly observed in Figure 2c. Additionally, the electrodes’ height decreased from 15 µm to 4.42 µm, according to confocal microscopy measurements, corresponding to a 70% reduction. The electrical resistivity of the resulting GC structures averaged to 1.34 × 10^−4^ Ωm.

### 3.2. Microalgae Preparation and Analysis

A culture of *Neochloris oleoabundans* 1185 (UTEX, Austin, TX, USA) was maintained for 15 days in Modified Bold 3 N medium to increase biomass. Then, to obtain HLC and LLC cells, different cell samples were grown in a nitrogen-deplete (N−) and a nitrogen-replete (N+) medium of 8.82 mM and 0.1 mM NaNO_3_, respectively, under constant air bubbling. The room temperature was maintained at 27 °C and 55% relative humidity, and the samples were exposed to 18 h-light/6 h-dark photo periods using a white fluorescent lamp for 11 days. To monitor cell growth, the optical density of the samples was measured by spectrophotometry at 750 nm, OD_750_ (VersaMax, Molecular Devices, Sunnyvale, CA, USA). In this case, two measurements were taken for each sample and the results were averaged.

To measure cell lipid content, the Nile Red protocol was followed [44]. Here, a 5 µL microalgae sample with an OD_750_ = 3.0 was mixed with 50 µL of dimethyl sulfoxide (DMSO). The mixture was vortexed and then heated in a microwave oven at 800 W for 60 s. Next, 10 µL of Nile Red solution (Sigma Aldrich, St. Louis, MO, USA) in acetone (100 μg/mL), and 935 µL of MilliQ water type I (Merck Millipore, Billerica, MA, USA) were added to a 1 mL volume of the microalgae sample and vortexed again for homogenization. The resulting mixtures were heated and vortexed for 60 s and, after 10 min of resting for a better penetration of the dye, the fluorescence intensity was measured with a microplate reader (Gemini XPS, Molecular Devices, Sunnyvale, CA, USA) with excitation and emission wavelengths of 530 nm and 590 nm, respectively. Four replicates were used for every sample. Results from the spectrometry analysis to monitor cell growth, and the fluorescence analysis to determine lipid content in cells, were plotted and statistically analyzed with an unpaired *t*-test, considering *p*-values of 0.05 and 0.01.

Once biomass under N− medium reached higher lipid accumulation than biomass under N+ medium (at day 11), 2 mL samples with OD_750_ = 3.0 were separately centrifuged to discard the supernatant, and DEP solution was added to the pellet remains. This procedure was carried out three consecutive times to avoid conductivity changes in the DEP solution (the conductivity of culture medium is higher than that of the DEP solution). The DEP solution consists of deionized water with potassium phosphate to an ionic conductivity of $\sigma_{m}=30$ μS/cm, measured using a benchtop conductivity meter by immersing the measuring electrode into 30 mL of DEP solution moments before the experiments were carried out. For osmotic equilibrium, which was required to avoid significant pressure on the cells, 3 g/L of glucose was added without significant observable effect on conductivity of the solution [38]. Prepared samples were pipetted into the microfluidic channel and the AC signal was applied across the electrodes. The amplitude of the signal was fixed to 7 V_PP_ and the frequency was swept from 100 kHz to 30 MHz. For experiments carried out in the kHz range, the frequency was swept in 100 kHz steps. Moreover, for experiments carried out in the MHz range, the frequency was swept in 5 MHz steps since no observable effects were noticed for narrower frequency steps. The induced DEP motion of the cells was observed with an inverted microscope (Nikon Eclipse LV100, Nikon Instruments, Melville, NY, USA) and a digital camera (SPOT RT-KE, Diagnostic Instruments, Inc., Sterling Heights, MI, USA). Each experiment was observed for 15 s, to ensure a steady state of cell distribution due to DEP effects; however, the transient state of cells (cell movement due to electrostatic balancing due to the AC potential across the microchannel) typically occurred in the first 5 s after the AC signal was applied.

## 4. Results

### 4.1. Microalgae Growth

**Figure 3:**
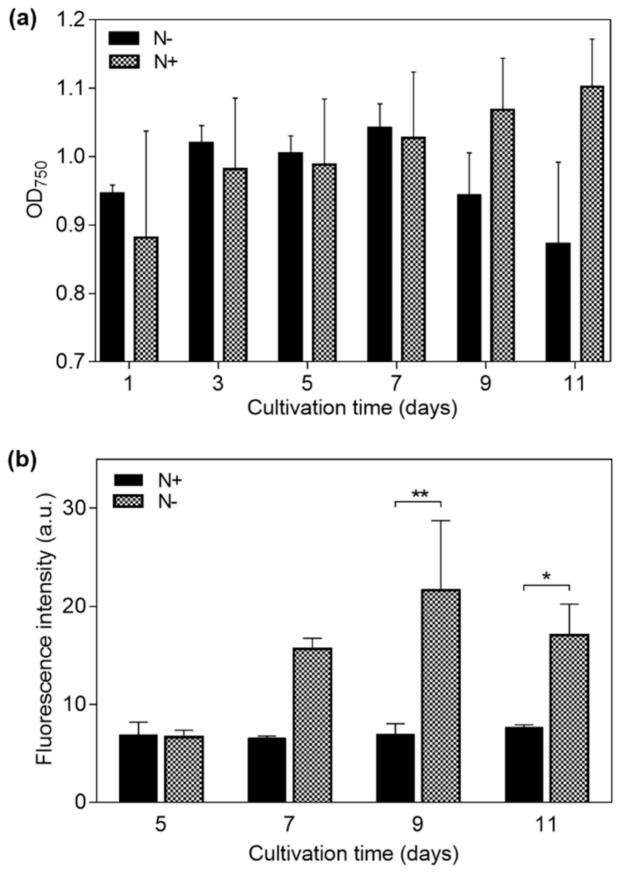
Measurements on *Neochloris oleoabundans* samples cultured under nitrogen-replete (N+) and nitrogen-deplete (N−) medium conditions for 11 days. (**a**) Optical density at 750 nm (OD_750_) for cell density in the suspending medium, and (**b**) fluorescence intensity indicating cell lipid content for days 5, 7, 9, and 11 (*p*-values: * ≤ 0.05 and ** ≤ 0.01).

Microalgae cultured under the N− condition showed a mean decrease in biomass concentration at day 11 of 7.82%, relative to day 1. In contrast, microalgae under the N+ condition increased 24.5% in biomass concentration at the same day, as shown in Figure 3a.

A fluorescence intensity plot for the N+ and N− samples is shown in Figure 3b. An increase in fluorescence intensity of N− cells started to develop at day 7, indicating a significant cytoplasmic lipid content gain. In contrast, the fluorescence intensity of N+ cells remained constant throughout the days of cultivation, indicating a relatively constant lipid content for this sample. The maximum fluorescence intensity for N− cells was registered at day 9—310% higher than that which was obtained for N+ cells on the same day. Finally, at day 11, N+ cells showed a slight increment of 15% in comparison to day 1; however, N− cells showed a fluorescence intensity that was 180% higher than the N+ sample. A significant difference (unpaired *t*-test) between the N+ (containing LLC cells) and N− (containing HLC cells) samples was found after day 7, as indicated in Figure 3b, supporting the effectiveness of the selected strategy for lipid accumulation in the studied microalgae.

### 4.2. Trapping Zones Estimation

**Figure 4:**
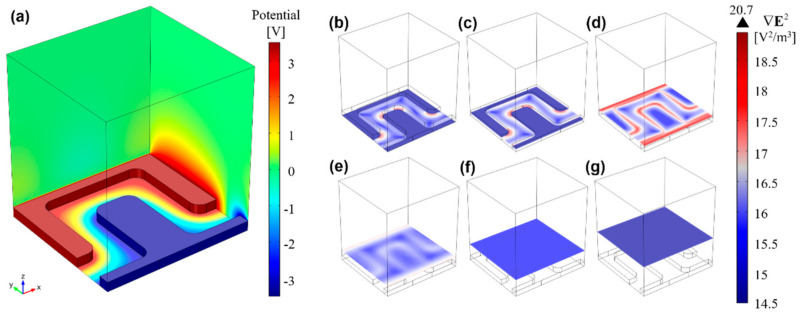
3D finite element analysis of (**a**) the electric potential distribution (V), gradient magnitude of the electric field squared, $\nabla \left(E\cdot E\right)$ (logarithmic scale), and trapping regions at different *xy*-planes: (**b**) 0 μm; (**c**) 2.5 μm; (**d**) 5.5 μm; (**e**) 9.5 μm; (**f**) 15.5 μm; (**g**) 27.5 μm. From these results, it is foreseen that microalgae experiencing pDEP will be drawn towards the outward electrode corners and edges (highest $\nabla \left(E\cdot E\right)$ regions), whereas those cells undergoing nDEP will be projected towards upper microchannel planes (lowest $\nabla \left(E\cdot E\right)$ region).

Figure 4a shows the electric potential distribution, *φ*, across the DEP solution for a potential difference of 7 V_PP_ across the electrodes, including the substrate/solution interface (domain 3 in Figure S1 and parameter details shown in Table S1), where a linear potential drop is observed. Figure 4b–g shows different *xy*-plane cuts along the channel height for $\nabla \left(E\cdot E\right)$. At the bottom of the channel (Figure 4b), pDEP trapping zones can be observed in red at electrode corners, and an increase in magnitude along the electrode height (Figure 4c), reaching maximum magnitude at the electrodes top plane (Figure 4d). At the top plane, pDEP trapping is also expected to occur at the electrode borders, and not only at the corners. For higher plane cuts, $\nabla \left(E\cdot E\right)$ rapidly decreases (Figure 4e,f), until becoming negligible at 27.5 µm from the bottom (Figure 4g).

### 4.3. DEP Differentiation of Microalgae Lipid Content

For a comprehensive assessment of the DEP effect on LCC and HLC cells, we carried out experimental tests over a wide frequency window (from 100 kHz to 30 MHz) and the results were split into three ranges: (*i*) low-frequency range: 100–800 kHz; (*ii*) mid-frequency range: 1–10 MHz; (*iii*) high-frequency range: 15–30 MHz.

**Figure 5:**
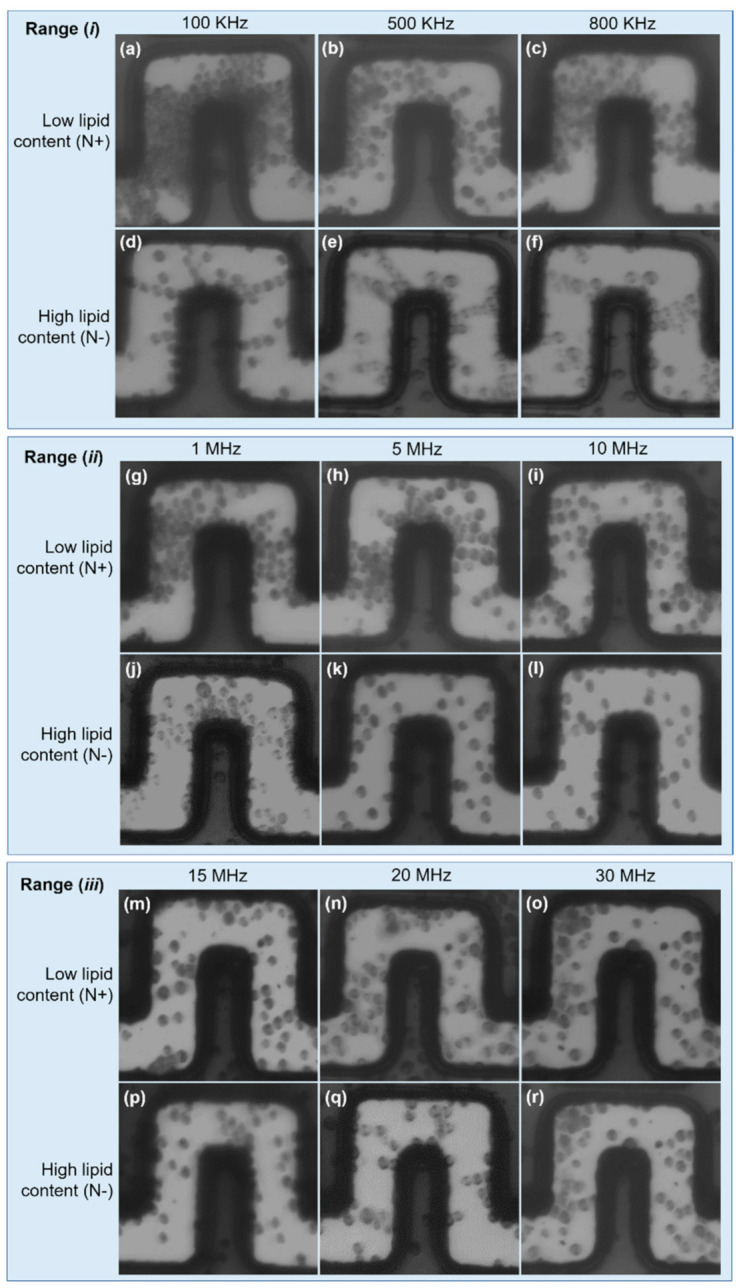
Characterization of HLC and LLC cells. Low frequency range: (**a**–**f**). A strong pDEP effect is experienced by LLC cells. In contrast, slight pDEP attractions were noticed in HCL cells, demonstrated by pearl chain formations due to weak cells polarization. Mid frequency range: (**g**–**l**). pDEP is experienced by LLC cells at 1 MHz and 5 MHz, and becomes negligible at 10 MHz. HLC cells show nDEP at 5 and 10 MHz. High frequency range: (**m**–**r**). Both cell types experience nDEP, which drives the cells to low $\nabla \left(E\cdot E\right)$ magnitudes. Cell fouling can be appreciated in all cases.

In (*i*), LLC cells undergo strong pDEP, causing large cell aggregation at the front and lateral electrode edges, and clear areas at inward electrode corners (i.e., trapping zones determined by the FEA). This effect is dominant at 100 kHz and weakens as the frequency increases, as can be seen in Figure 5a–c. HLC cells sustain a weak pDEP effect, only manifested by pearl chain formations due to dipole–dipole interactions among neighboring cells. Evidence of this behavior is shown in Figure 5d,e, whereas cell polarization observations became inconclusive at 800 kHz (Figure 5f), also suggesting a decreasing pDEP effect as the frequency is increased.

The driving pDEP force decreased for both cell types in (*ii*); however, LLC cells still undergo weak pDEP at 1 and 5 MHz, becoming negligible at 10 MHz, which defines the upper frequency limit of pDEP on these cells as between 5 and 10 MHz (Figure 5g–i). Regarding HLC cells, from range (*i*) to range (*ii*), pearl chain formations broke down to single cells, suggesting the depletion of pDEP effects at the mid-frequency range. This breakdown can be clearly seen following the transition from Figure 5e (500 kHz) to Figure 5f (800 kHz), Figure 5j (1 MHz), and Figure 5k (5 MHz). Furthermore, some of these cells now seem to be subjected to nDEP at 5 and 10 MHz, based on the low number of cells found across the electrode gaps, likely due to fouling (Figure 5k,l). In this case, most of these cells were driven towards upper channel regions, which were identified as nDEP regions from the FEA.

For a further increase in frequency (*iii*), neither HLC nor LLC cells were aggregated at pDEP trapping regions, as shown in Figure 5m–r. This observation suggests that both LLC and HLC cells undergo nDEP, and the cells are driven toward low $\nabla \left(E\cdot E\right)$ zones. The observed cells in Figure 5m–r are possibly stacked on the substrate surface due to fouling effects.

**Figure 6:**
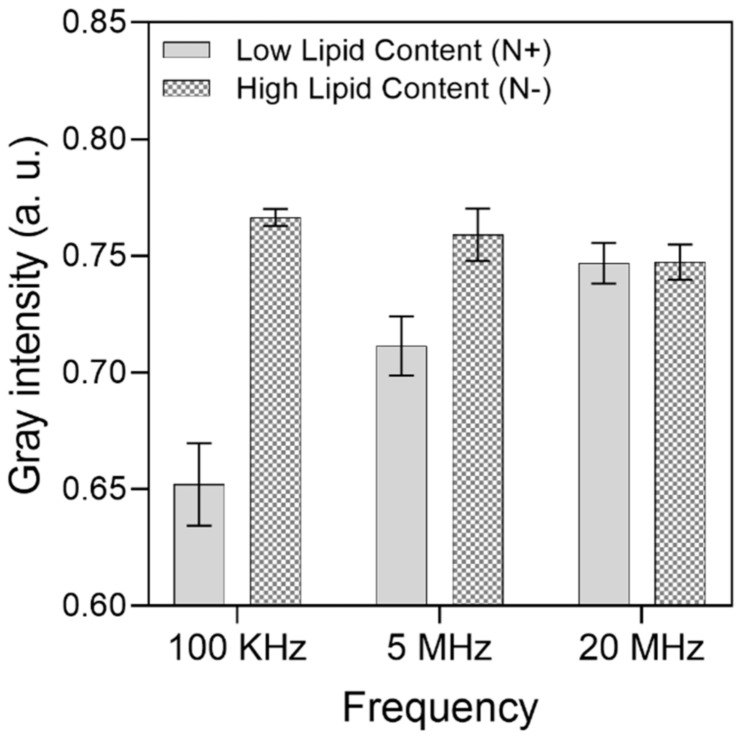
Gray intensity analysis of microalgae cell differentiation between high and low lipid content cells. A total of 256 gray values were considered from a range of 0 (black) to 1 (white). For HLC cells at 100 KHz, mean = 0.76 and standard deviation (SD.) = 0.003. For LLC cells at 100 KHz, mean = 0.65 and SD. = 0.01. For HLC cells at 5 MHz, mean = 0.75 and SD. 0.01. For LLC cells at 5 MHz, mean = 0.71 and SD. = 0.01. Finally, for HLC cells at 20 MHz, mean = 0.74 and SD. = 0.007. For LLC cells at 20 MHz, mean = 0.74 and SD. = 0.008.

A gray intensity analysis was carried out to quantitatively differentiate the cell aggregation between the electrodes among the HLC and LLC samples. For each frequency range, 10 different trapping zones (electrode gaps) across the IDEA were assessed individually; then, the widest intensity difference was considered, and the results are presented in Figure 6. Here, the lower the intensity, the higher the number of cells accumulated between the electrodes due to the obstruction of light bouncing from the substrate to the microscope objective. From this analysis, the widest intensity difference between HLC and LLC cells is 100 kHz, which supports the results presented on the optical images. For 100 KHz, the gray intensity of LLC cells averaged at 0.65, with a standard deviation of 0.01, whereas for HLC cells, the gray intensity averaged at 0.76, with a standard deviation of 0.003. This suggests that considerably more HLC cells experience pDEP at this frequency, and most of the sampled trapping zones consistently capture around the same amount of HLC cells. For the 5 MHz frequency, the mean and standard deviation for HLC cells was 0.75 and 0.01, and 0.71 and 0.12 for LLC cells, respectively. Finally, for the 20 MHz frequency, the mean and standard deviation for HLC cells was 0.74 and 0.007, and 0.74 and 0.008 for LLC cells.

## 5. Discussion

In this study, *Neochloris oleoabundans* microalgae with high and low lipid contents were characterized by their DEP response when stimulated with an AC amplitude of 7 V_PP_, and frequencies ranging between 100 kHz and 30 MHz using a glassy carbon castellated IDEA. With the set-up presented here, promising results were demonstrated regarding the characterization of microalgae with high and low lipid content using a low-amplitude AC signal on a wide frequency range.

Both LLC and HLC are highly polarizable and experience dielectrophoretic forces. LLC cells exhibited pDEP when stimulated on frequencies ranging between 100 kHz and 5 MHz. In contrast, HLC cells experienced nDEP on frequencies ranging from 1 to 30 MHz. This suggests that the cells possess a “dielectrophoretic fingerprint”, which is a function of cell lipid content and applied voltage frequency.

The cytoplasmic lipid content determines the complex permittivity of the cells, which allows the cells to be either driven towards or rejected from the high-intensity electric field regions. In our study, we observed that LLC cells possess more inner charges than those on the fluid side boundary, making $f_{CM}$ positive. Thus, the cells undergo pDEP, driving them towards the electrode edges (the high intensity electric field region). For the case of HLC cells, as they possess lower inner charges than those on the surrounding fluid, $f_{CM}$ becomes negative. Thus, the cells undergo nDEP, which rejects these cells from high-intensity electric field regions.

A frequency window between 1 and 5 MHz was identified as the frequency range where HLC and LLC cells undergo opposite DEP effects (i.e., pDEP for LLC cells and nDEP for HLC cells) for a fluid with a conductivity of $\sigma_{m}=30$ μS/cm. This contrast in DEP effects between LLC and HLC cells, allows LLC cells to be filtered out from the sample, producing an HLC-rich sample that could be easily recovered afterwards.

The intensity analysis provides an indirect quantitative measure of cell accumulation at the electrodes gap. This measure reveals a trend that suggests that the strongest differentiation between HLC and LLC cells takes place at 100 kHz and reduces as the frequency increases. From the analysis of the results, we found that, although the strongest visual differentiation occurred at 100 kHz, both LLC and HLC cells undergo pDEP, which might cause LLC-contaminated samples for recovery. In this sense, the frequency window between 1 and 5 MHz (where LCC and HLC cells experience opposite DEP forces) is the range that could be used to highly purify the HLC sample for recovery.

## 6. Conclusions

The use of Carbon-DEP for the rapid screening of microalgae lipid content might help optimize the harvesting time in large-scale microalgae production by efficiently and economically analyzing low sample volumes of microalgae to determine their optimum lipid content, based on their dielectrophoretical characterization. The use of the Carbon-MEMS process for electrode fabrication also represents a cost-effective alternative to conventional and costly technologies using on other materials (typically noble metals), without sacrificing performance and reliability. We believe that, due to the simplicity of the Carbon-MEMS and soft-lithography processes, our approach can be scaled for the mass production of Carbon-DEP-based microfluidic devices. Finally, the intrinsic chemical properties of glassy carbon electrodes, including excellent biocompatibility and a wide electrochemical stability window, suggest that this material can be used to characterize the DEP response of a wide variety of living cells in a label-free fashion.

## Supplementary Materials

The following are available online at https://www.mdpi.com/article/10.3390/mi12091023/s1, Figure S1: Periodic section of the interdigitated electrode array used for the finite element analysis. Dimensions are shown in black and boundaries in red. ***E***1 and ***E***2 correspond to electrodes of opposite combs, Table S1: Boundary conditions applied in the domain in Figure 2. V0 = 7 VPP, SRC = source boundary and DST = destination boundary.
