# Supplementary material for: Rapid Lipid Content Screening in Neochloris oleoabundans Utilizing Carbon-Based Dielectrophoresis
Source: Micromachines (Basel). 2021 Aug 27;12(9):1023. doi: 10.3390/mi12091023 (PMC8471556; doi:10.3390/mi12091023)
Supplement: Supplementary file 1 [file micromachines-12-01023-s001.zip › micromachines-1320598-supplementary.pdf]

micromachines

Supplementary Materials

Rapid Lipid Content Screening in *Neochloris Oleoabundans* Utilizing Carbon-Based Dielectrophoresis

Cynthia M. Galicia-Medina, Matías Vázquez-Piñón, Gibran S. Alemán-Nava, Roberto C. Gallo-Villanueva, Sergio O. Martínez-Chapa, Marc Madou, Sergio Camacho-León, Jonathan S. García-Pérez, Diego Esquivel-Hernández, Roberto Parra-Saldívar and Víctor H. Pérez-González

The FEA was carried out using COMSOL Multiphysics (COMSOL, Inc., Stockholm, SE) to analyze the trapping zones across the microfluidic channel either by pDEP or nDEP. For this purpose, a 3D geometry of a periodic section of the microelectrode array was established. The geometric domain of the model, including domain boundaries is described in Figure SI.1, and the respective boundary conditions in Table SI.1. The model was solved to find the electric field distribution across the microfluidic channel by applying a potential difference of 7 VPP across the electrodes, and E2 was subsequently calculated. The fluid filling the area corresponding to the microchannel was modeled with an electrical conductivity of 30 μS/cm and a relative permittivity of 78, corresponding to the experimental solution. The electrodes were defined as ideal conductors, thus ohmic losses were neglected.

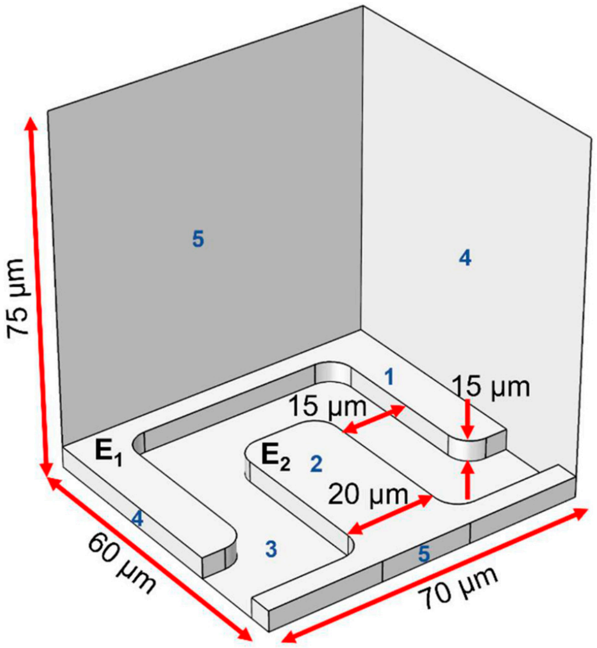

Figure S1. Periodic section of the interdigitated electrode array used for the finite element analysis.Dimensions are shown in black and boundaries in red. E1 and E2 correspond to electrodes of opposite combs.

Table S1. Boundary conditions applied in the domain in Figure 2. V0 = 7 VPP, SRC=source boundary and DST=destination boundary.

Boundaries	Boundary condition	Equation
1	Electric potential 1	$V = V0$
2	Electric potential 2	$V = 0$
3	Electrical insulation	$\mathbf{n}\cdot\mathbf{J} = 0$
4	Periodic condition 1	$VSRC1 = VDST1$
5	Periodic condition 2	$VSRC2 = VDST2$
